# The Contribution of Willingness to Communicate to L2 Learners’ Depth of Vocabulary Knowledge: An Empirical Study

**DOI:** 10.1007/s10936-024-10062-z

**Published:** 2024-04-08

**Authors:** Kamal Heidari

**Affiliations:** Department of Linguistics and Applied Language Studies, Victoria University, Wellington, New Zealand

**Keywords:** WTC, Depth of vocabulary knowledge, WAT, WPLT, Triangulation, L2 Learners

## Abstract

The issues of depth vocabulary knowledge and Willingness to Communicate (henceforth, WTC) are among the most important issues in second language learning. The present study set out to empirically look into the contribution of WTC to depth of vocabulary knowledge in L2 learning. To this end, 88 English L2 learners, divided into two groups in terms of their WTC, were given two depth vocabulary tests. The Word Association Test (WAT) was first administered to make a comparison between the depth vocabulary knowledge of the two WTC groups. Then, to triangulate the results, the Word Part Levels Test (WPLT) was administered to check whether the obtained results confirmed those of WAT. Analyzing data through independent t-test and MANOVA indicated that learners with higher levels of WTC had deeper vocabulary knowledge than those with lower levels of WTC on the WAT. Further, the triangulation results evinced that although the two groups did not differ significantly on the form-section and meaning-section of the WPLT, they significantly differed on the use-section of the test. The relevant pedagogical implications of the study are discussed.

## Introduction

Vocabulary is among the most pivotal aspects of language (Heidari, 2015; Laufer, 2017). Studies on L2 vocabulary acquisition have substantial effects on informing language education (Masrai, 2021). As such, vocabulary has been examined extensively by scholars and consequently, insightful findings have been gained, which have, in turn, clarified many ambiguous points associated with vocabulary (Heidari, 2019). However, despite these achievements, there are still areas that require further investigation. This is because of the multidimensional quiddity of vocabulary (Nation, 2022; Schmitt et al., 2001). Vocabulary consists of a number of components and sub-components that take time and effort to learn. One dimension of vocabulary that is still underresearched is the issue of depth vocabulary knowledge (Gonzalez-Fernandez & Schmitt, 2019; Laufer & Goldstein, 2004; Read & Dang, 2022). It simply refers to how well an L2 learner knows a lexical item. Depth of vocabulary is important as it assists L2 learners with implementing communicative tasks more efficiently (Yanagisawa & Webb, 2020). Having deep knowledge about a lexical item strengthens both receptive and productive competency of learners. It improves receptive competency because learners with deeper vocabulary command can reach a better and faster understanding of a piece of language than learners with less depth of vocabulary command. Additionally, learners who possess deeper vocabulary knowledge can produce language more effectively, compared with those with less depth of vocabulary knowledge, simply because the former group of learners know the subtle context-specific properties of lexical items, which aid them to produce language more contextually appropriate than the latter group of learners. That said, it is important to examine factors that might help L2 learners to deepen their vocabulary knowledge. One of these factors is individual differences individual factors (Dörnyei, 2009), specifically WTC (Hadley, 2018; Laufer, 2017; Zhong, 2012).

WTC is typically depicted as the degree to which one is inclined to be engaged in a particular communicative event (Wood, 2016). It is considered to constitute a direct antecedent to communication (Ducker, 2022, p. 216) playing a pivotal role in accounting for learners’ success in L2 acquisition. Given its importance, a great deal of research has been conducted, especially in recent decade, on the interaction of WTC with learning of varying aspects of language. It seems that a considerable bulk of these studies have focused on the contribution of WTC to other individual differences factors, such as anxiety, grit, motivation, to name a few. WTC, however, play an integral role in enhancing learners’ competency of language skills and domains, an issue that has surprisingly been overlooked. Zarrinabadi (2021) similarly called for further research on WTC in association with other language skills and domains (p. 261). One domain that needs to be further researched is the contribution of WTC to vocabulary, specifically depth of vocabulary knowledge. There is little empirically recognized understanding as to whether L2 learners’ WTC and depth vocabulary knowledge interact. This research study, then, aims to bridge this gap by exploring the interaction of WTC and depth of vocabulary knowledge among L2 learners. The study specifically addressed the following research question:

- Does L2 learners’ WTC contribute to their depth of vocabulary knowledge? If yes, how?

The study is of great importance in that its findings can inform L2 instruction. To put it another way, in case the study finds a positive contribution for WTC to depth of vocabulary knowledge, language teachers can adopt those teaching strategies and methods that increase learners’ WTC and, consequently, deepen their vocabulary command.

## Depth of Vocabulary Knowledge

Possessing a sizable repertoire of vocabulary is an important factor for successful language learning (Laufer & Nation, 1995; Van Goch et al., 2018; Yu, 2010). Depth/breadth vocabulary knowledge is a prominent strand playing a core role in the ultimate performance of learners. Breadth of vocabulary is simply defined as the total number of words a learner knows (Hadley & Dickinson, 2018) while depth of vocabulary knowledge refers to how well a learner knows a word (Mckeown & Beck, 2014; Qian, 2002). This definition cannot convey the precise nature of depth knowledge. Similarly, Milton (2009) also conceded that there has not yet been offered any comprehensive definition for the depth of vocabulary knowledge.

While breadth of vocabulary knowledge is unidimensional (referring simply to form-meaning knowledge of a word) and easy to understand, depth of vocabulary knowledge is multidimensional (it includes varying sub-components) and hard to grasp. Manyak (2011) highlighted the intricate nature of depth of vocabulary knowledge arguing that it is a kind of metalinguistic awareness. By metalinguistic awareness, it means conscious knowledge about language in which language is a tool of inquiry not an object of study. In line with metalinguistic awareness definition, for depth of vocabulary knowledge, learners endeavor to raise their awareness of all possible meaning layers of a lexical item, including when, where, and how a lexical item can be used. Depth knowledge is, in fact, the sum of knowledge about varying interrelated components involving spoken and written form, morphological, social, connotative, and associational knowledge (Laufer & Goldstein, 2004).

Different accounts and classifications have been offered for depth of vocabulary knowledge. Manyak (2011), for instance, enumerated three types of knowledge embedded in depth knowledge: morphology (knowledge about different parts of a word as well as its orthographic form), semantics (knowledge about meaning(s) of a word), and syntax (knowledge about where and how a word should be used to be grammatically appropriate). This taxonomy, however, does not reflect a complete picture of depth knowledge, in that some important aspects, such as pragmatic features (in what social cultural contexts a lexical item should or should not be used), have not been considered. Highlighting that depth of vocabulary knowledge refers to the multiple layers of meaning for a single lexical item, Nassaji (2004) clarified the concept of depth of vocabulary knowledge by involving more fine-grained components to depth vocabulary knowledge, namely, knowledge about spelling, pronunciation, register, morphological and stylistic features, as well as syntactic and semantic relationships of words with other words, and knowledge of antonym, synonymy, and hyponymy.

**Fig. 1:**
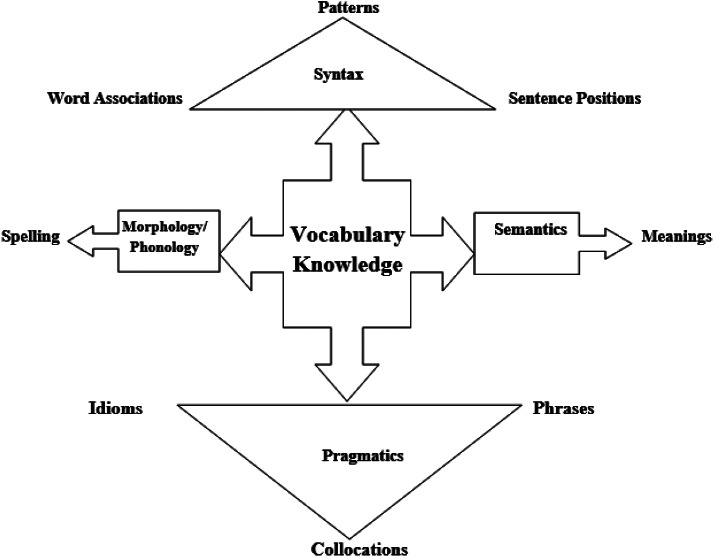
Dimensions of vocabulary knowledge

Despite the valuable information that the above explanations and classifications offer, it is still hard to picture vocabulary knowledge clearly. The researcher has used Fig. 1 to raise learners’ awareness of vocabulary multi-dimensionality and has found it effective. It is, then, brought here so that others may use it to help their learners to grasp the nature of vocabulary clearly. When learners understand that vocabulary knowledge is not merely a form-meaning knowledge but entails different dimensions, they will not limit their word learning to considering the form-meaning connection anymore and will attempt to increase their knowledge about other aspects of words as well. In this figure, the spelling and meaning dimensions are pertinent to breadth knowledge of vocabulary, while other dimensions pertain to depth knowledge.

The first dimension is morphology/phonology. It simply refers to how a word is spelled and articulated. As an example, morphology/phonology knowledge of the word “enjoy” means that learners know that this word consists of a string of “e, n, j, o, and y” letters and two syllables of “en” and “joy”. The pronunciation is also involved in the same facet. The second dimension is the semantic part that specifies all possible meanings of a word. Regarding the word “enjoy”, learners should know that the word “enjoy” means “to feel happy about something or somebody” as the primary meaning and “to have the benefit of” as the secondary meaning. The third dimension pertains to syntactic features of words. It embraces different sub-components such as grammatical patterns (for example “enjoy” requires no preposition), sentence positions (“enjoy” should be used after the subject followed by gerund), and word associations (paradigmatic and syntagmatic relationships). Finally, the fourth dimension, which is tightly related to syntax dimension, is the pragmatic aspect. It is associated with how a word is used together with other components in a context. It incorporates a set of subcomponents the most important ones include idioms, phrases, and collocations.

Yanagisawa and Webb (2020) also proposed three approaches to measure depth of vocabulary knowledge: (a) developmental approach, which sees depth of vocabulary knowledge as developmental degree of word knowledge (from no knowledge to fully developed knowledge). The Vocabulary Knowledge Scale (VKS) by Paribakht and Wesche (1993) is an example of a scale that aligns with developmental approach (b) lexical network approach, which conceptualizes depth of vocabulary knowledge as a lexical network in mental lexicon. Read’s (1993) WAT is a test that accords with the features of this approach and (c) component approach, which conceptualizes depth of vocabulary knowledge as knowing different derivative aspects of words. An example of a test that is in line with this approach is the WPLT by Sasao and Webb (2017) measuring knowledge of affixes as a component of vocabulary knowledge. Yanagisawa and Webb (2020) argued that researchers might consider one or more of these approaches in their studies depending on their research purposes. The present study considered the network approach and component approach and, accordingly, used the WAT and WPLT to measure learners’ depth knowledge, as these two approaches, compared with the developmental approach, have gained more popularity among researchers. Further, these two approaches assume that making use of words is closely pertinent to knowing them better. Macis et al. (2018) also assumed that using words in different situations is strongly related to gaining more knowledge about different components of them (p. 641).

As with empirical studies on depth vocabulary knowledge, most of them are pertinent to the relationship or impact of depth knowledge with/on skills, especially reading skill. They mostly reported a good positive predictive role for depth of vocabulary knowledge as far as language skills are concerned (Qian & Lin, 2020). As an example, Bolger et al. (2008) and Hadley et al. (2018) reported a positive role of depth vocabulary in reading skill. Zareva et al. (2005) found that depth of vocabulary knowledge was a significant indicator of proficiency level. Crossley et al. (2014) also looked into the effect of depth of vocabulary knowledge (specifically, association knowledge) on spoken and written language. Their study revealed that depth of vocabulary knowledge played a critical role in speaking and writing proficiency. Han (2017) and Qian (2002) also found depth of vocabulary knowledge a better predictor of receptive skills, compared with breadth of vocabulary. However, no study (to the best knowledge of the author) has ever dealt with the interplay of depth vocabulary knowledge and individual differences in general and WTC, in particular. This article, then, addressed this gap by inquiring into how WTC and depth vocabulary knowledge interact.

### WTC

When the ultimate purpose of learning a second language is communication, it stands to reason stating that pedagogy should be communication oriented as well. Pedagogical practitioners should draw on any theories, concepts, methods, and strategies that assist them with fostering learners’ communicative abilities. One concept that might be a determinant in achieving this goal is WTC (Suksawas, 2011). WTC simply refers to one’s inclination to initiate and engage in a meaningful communicative act (Cetinkaya, 2005; Heidari, 2019). Despite this simple definition, WTC is more complicated than it may appear, as many different factors have been reported to affect it. MacIntyre et al. (1998) proposed the oft-cited pyramid model depicting different linguistic, psychological, and contextual factors that may affect L2 WTC. Heidari (2019, p. 910) also presented a more pedagogical-based model in which factors affecting WTC have been divided into two main categories: self-related factors and other-related factors. The former is further subdivided into personality and proficiency factors while the latter includes context and teacher/peer immediacy.

In a very seminal article, MacIntyre (2012) elaborated two major constituents of WTC by referring to currents and waves metaphor. According to this model, currents refer to largely stable affective and behavioral patterns while waves refer to the surface variability of WTC that might change over time depending on situation. While both currents and waves are equally important to be considered in assessing learners’ WTC, researchers often consider one of them in their studies, due to varying reasons such as space restriction or lack of access to adequate facilities. Wood (2016), for example, measured WTC from waves perspective using specific tools and software. Although using both constituents in a single study adds to the validity of study, drawing on one constituent is also acceptable considering issues such ecological validity and practicality issues. That said, in the present study only the current component of WTC was considered to distinguish high and low WTC learners.

Another noteworthy point is that WTC can both be affected by and affect different factors. To name a few, Peng (2007) and Yu (2009) reported that group cohesiveness and classroom climate influence WTC. Freiermuth and Jarrell (2006) explored the impact of online chat on WTC. MacIntyre et al. (2002a) investigated the interrelationship of age and gender with WTC. Cao (2011) looked into the role of linguistic and pragmatic competence on WTC. Cao and Philp (2006) explored the effect of task type on WTC. On the other hand, studies have also found WTC may impact on other variables and aspects as far as language learning is concerned. For example, Dörnyei and Kormos (2010) investigated the impact of different factors including WTC on learners’ engagement in oral tasks. MacIntyre et al. (2003) examined the effect of WTC on learners’ performance in intensive language programs. Bergil (2016) looked into the impact of WTC on learners’ overall speaking skills.

Although the relevant literature has provided insightful findings on WTC, WTC still deserves further attention mainly due to its critical role in language learning process. One area that has been overlooked is the contribution of WTC to depth of vocabulary knowledge. Although it is generally argued that learners need to be engaged in language learning activities to foster their learning, no study, to the author’s best knowledge, has ever empirically investigated the contribution of WTC to depth of vocabulary knowledge. This study was, then, conducted to address this gap.

## Method

### Participants

The participants of the study were 88 young adult Iranian EFL learners (42 males and 46 females) with the age range of 18 to 23 years old. They all spoke Persian as mother language and had no experience of being in an English-speaking country. They were all sophomore college students in English Language Translation, English Language Teaching, and English Language Literature academic fields of study. Their general English language proficiency was determined to be upper intermediate by an Oxford Placement Test (OPT). The OPT (Allen, 2004) was utilized because it is reliable, objective, and easy to administer. Further, the test has been reported to have high validity; and its reliability through Cronbach alpha method was reported by Geranpayeh (2003) to be 0.90. The Cronbach alpha value obtained for the present study was 0.84. According to the test guidelines, the participants were upper intermediate L2 learners (scoring between 61and 80) corresponding to B2 level of the Common European Framework of Reference for Languages (CEFR). As with the LexTALE, it is a fast and practically feasible test to measure vocabulary command for mid to high proficient L2 learners. It comprises a simple lexical decision task. It is free, quick, easy to administer, and free, and has been reported as a valid and standardized test of vocabulary knowledge and a fair indication of general English proficiency (Lemhöfer & Broersma (2012). These two tests were decided to be used for assessing the participants’ vocabulary knowledge so that a more complete picture of their vocabulary command could be gained. The results of this test also confirmed the upper intermediate level of the participants (*M* = 71% and *SD* = 12.4).

Finally, the informed consent of the participants was also obtained; and to mitigate their negative feelings including stress, the researcher succinctly depicted the goals of the study to them and assured them that their performance on the tests would not affect their academic term performance.

### Instruments

Altogether, five instruments were utilized in this study. The first one was the OPT test administered among the participants to ensure their homogeneity in general English language knowledge level, including vocabulary. It consisted of 100 items testing varying language skills and domains. The test was administered in accordance with its guidelines and instructions. The second instrument was the LexTALE test for measuring the learners’ vocabulary knowledge.

The third instrument was the WTC questionnaire by Baghaei (2011) that has specifically been developed to measure the WTC degree of learners in EFL contexts. The questionnaire consists of 20 items each item requiring the learners to pinpoint their agreement to initiate communication in a given putative situation. They should respond to the items on a two-level scale (either agree or not). Therefore, the scoring procedure of the questionnaire is dichotomous in the sense that for agree responses 1 and for not agree responses 0 should be dedicated (Baghaei, 2011). The merit of this questionnaire over other questionnaires is that it has been developed specifically for EFL contexts and participants can imagine the situations more vividly. Having administered the questionnaire, 41 of the participants were specified to be High WTC L2 learners and 47 of them were determined to be Low WTC learners. Regarding the scale reliability, Cronbach alpha was calculated which turned out to be approximately 0.76. As with its validity, the questionnaire was double-checked by two statistics and testing experts and was confirmed to be appropriate for the current study purpose.

The fourth and fifth instruments were used to measure participants’ depth of vocabulary knowledge. First, the commonly-used Word Association Test (WAT) by Read (1993, 1998a, b, 2000) was given to the participants. This test measures depth of vocabulary knowledge via word associations. It comprises 40 items each having a stimulus word followed by four words in the left box (often synonyms) and four ones in the right box (words that commonly follows the stimulus). The participants are expected to choose four of these eight options as correct answers. To score it, each correct choice receives one point. Therefore, the maximum score is 160 for the 40 items. A sample of the test is as follows:

### Sound

**Table:** 

**(a) Logical (b) Healthy (c) Bold (d) Solid**	**e) Snow f) Temperature g) Sleep h) Dance**

This test considers paradigmatic, syntagmatic, and analytic relations among lexical items. Thus, it can be claimed to be an appropriate tool for measuring the vocabulary depth knowledge (Akbarian, 2010; Qian, 2002). Further, the WAT is an example of the lexical network approach (Yanagisawa & Webb, 2020). Its reliability and validity have been repeatedly approved (Nassaji, 2004; Read, 1993, 2000). The Cronbach alpha formula ran for the test was 0.89.

After the WAT, the researcher used the intermediate version of the Word Part Levels Test (WPLT) of Sasao and Webb (2017). It contains 79 selection-type items in three sub-sections: form section (consisting of 37 items about prefixes and suffixes of English), meaning section (including 21 items about meaning of word parts), and use section (entailing 21 items about part of speech of word parts). This test was used to triangulate the results obtained from the WAT so that more conclusive overall conclusions could be made about the contribution of WTC to depth vocabulary knowledge. Triangulation can reinforce the reliability and validity of findings of a study (Baker & Egbert, 2016), as it provides a more comprehensive picture of a specific situation (Tashakkori & Teddlie, 1998). Unlike the WAT, which was in line with the lexical network approach, the WPLT is an example of components approach (Yanagisawa & Webb, 2020). Using these two tests in the present study provided a more complete picture of the participants’ depth of vocabulary knowledge.

### Data Collection/Analysis Procedure

Data collection took four sessions. In the first session, the learners were asked to take the OPT and LexTALE. Then, in the second session, they sat for the WTC questionnaire. Having determined their WTC level and divided them into High and Low WTC groups, in the third session, the two groups sat for the WAT test. Finally, in the fourth session, the WPLT test was administered among the participants. The time interval between the sessions was four days. Prior to giving the tests, the researcher briefly explained the tests and the way the learners should take them. Appropriate time limit was also set considering the number of items in line with the tests’ instructions. Furthermore, to analyze gathered data, descriptive statistics, independent t-test, and MANOVA were employed. Independent t-test was used for the OPT results and ensuring about homogeneity of the participants as well as for analyzing the WAT results. MANOVA was also run to examine the interrelationship of each of the sub-sections of the WPLT as a dependent variable and the WTC as the independent variable.

## Results

The study aimed to explore whether or not high and low WTC learners vary significantly in terms of depth vocabulary knowledge. An independent t-test was first run at the beginning of the study for analyzing the OPT results and checking whether there was any significant difference between high and low WTC groups (pertinent tables available in the supplementary file). The results demonstrated that the difference between the High WTC and Low WTC groups was not statistically significant (t = 0.75. Sig.≥0.05) and there was no considerable difference between the mean score of the two groups at the beginning of the study (59.62 vs. 55.84, respectively).

After ensuring about the homogeneity of the participants, an independent t-test was run to analyze the two groups’ results on the WAT. Table 1 shows the descriptive statistics of the test.

**Table 1:** Descriptive statistics of WTC and WAT depth vocabulary knowledge

	WTC	N	Mean	Std. Deviation	Std. Error Mean
Depth Knowledge	High WTC	41	124.6	19.2	3.3
	Low WTC	47	87.5	14.5	2.8

In this table, the High WTC group mean (*M* = 124.6) was considerably higher than the Low WTC group mean (*M* = 87.5) on the depth vocabulary knowledge test. To check whether this difference was statistically significant, Table 2 representing the main results of independent t-test should be checked.

**Table 2:** Independent t-test for WTC and depth knowledge

		Levene’s Test for Equality of Variances		t-test for Equality of Means						
		F	Sig.	t	df	Sig. (2-tailed)	Mean Difference	Std. Error Difference	95% Confidence Interval of the Difference	
									Lower	Upper
Depth Knowledge	Equal variances assumed	4.6	0.03	8.1	86	0.000	37.0	4.5	27.9	46.2
	Equal variances not assumed			8.3	85	0.000	37.0	4.4	28.1	45.9

The table demonstrates that the difference between the High WTC and Low WTC groups was statistically significant (Sig. ≤ 0.05). That is to say, WTC significantly contributed to the depth vocabulary knowledge of the L2 learners.

In the next stage, a MANOVA was run to analyze the data gathered from the WPLT. Having checked the related presumptions, such as normality and homogeneity of variations, Table 3 presents the underlying output of the MANOVA. In this table, the Wilks’ Lambda and Sig. values were 0.081 and 0.000, respectively, which indicated a significant difference between high and low WTC groups in their overall score on the WPLT.

**Table 3:** Multivariate tests on WTC and WPLT scores

Effect						Value		F		Hypothesis df			Error df		Sig.
Intercept			Pillai’s Trace			0.850		9125.112		3.000			84.000		0.000
			Wilks’ Lambda			0.012		9125.112		3.000			84.000		0.000
			Hotelling’s Trace			320.203		9125.112		3.000			84.000		0.000
			Roy’s Largest Root			320.203		9125.112		3.000			84.000		0.000
WTC			Pillai’s Trace			0.903		101.402		3.000			84.000		0.000
			Wilks’ Lambda			0.081		101.402		3.000			84.000		0.000
			Hotelling’s Trace			10.901		101.402		3.000			84.000		0.000
			Roy’s Largest Root			10.901		101.402		3.000			84.000		0.000

Table 4, revealing the interrelationship of each of the sub-sections of the WPLT as a dependent variable and the WTC as the independent variable, indicates that while the High and Low WTC groups did not significantly differ in their scores on form-section and meaning-section of the WPLT, they significantly differed in terms of their scores on the use-section of the test. Furthermore, the table also reveals that since the Partial Eta Squared values of the use-section is 0.831, according to Cohen’s criteria, WTC had a large impact on the use-section scores of the two groups.

**Table 4:** Tests of between-subjects effects for WTC and Depth vocabulary knowledge

Source	Dependent Variable	Type III Sum of Squares	df	Mean Square	F	Sig.	Partial Eta Squared
Corrected Model	FormSection	104.103	1	102.782	95.851	0.000	0.222
	MeaningSection	111.501	1	129.981	101.909	0.000	0.304
	UseSection	267.745	1	288.391	297.890	0.000	0.831
Intercept	FormSection	10611.403	1	12888.142	13451.225	0.000	0.431
	MeaningSection	11032.109	1	11466.722	11501.786	0.000	0.409
	UseSection	10254.200	1	10331.201	10738.920	0.000	0.442
WTC	FormSection	104.103	1	102.782	95.851	0.000	0.222
	MeaningSection	111.501	1	129.981	101.909	0.000	0.304
	UseSection	267.745	1	288.391	297.890	0.000	0.831
Error	FormSection	43.402	86	1.003			
	MeaningSection	38.332	86	0.701			
	UseSection	48.105	86	0.698			
Total	FormSection	11613.000	86				
	MeaningSection	10762.000	86				
	UseSection	09095.000	86				
Corrected Total	FormSection	123.876	87				
	MeaningSection	142.098	87				
	UseSection	287.285	87				

Lastly, the estimated marginal means table (Table 5) showed that the means of the two groups on form-section and meaning-section of the test were almost similar with a slight (but not significant) outperformance for the High WTC group.

**Table 5:** Estimated marginal means related to WPLT sub-sections and WTC

Dependent Variable	WTC	Mean	Std. Error	95% Confidence Interval	
				Lower Bound	Upper Bound
FormSection	1.00 High WTC	29.391	0.122	14.222	16.612
	2.00 Low-WTC	28.649	0.101	13.703	14.986
MeaningSection	1.00 High WTC	18.041	0.155	10.504	12.466
	2.00 Low-WTC	17.877	0.144	10.451	11.730
UseSection	1.00 High WTC	17.147	0.181	11.381	12.177
	2.00 Low-WTC	14.339	0.164	8.903	9.103

Collectively, analyzing the gathered data demonstrated that High WTC learners had deeper vocabulary knowledge than the Low WTC ones on both the WAT and WPLT, especially their use sections.

## Discussion

The results of the WAT confirmed that high WTC learners had a deeper degree of lexical knowledge than low WTC ones. Furthermore, the results of the WPLT demonstrated that the high WTC group had deeper lexical knowledge than the low WTC group on the form and meaning sections (not statistically significant) as well as use-section (statistically significant).

On the one hand, there has been extensive evidence that WTC plays a key role in L2 learning (Kim, 2004), especially given the recognized importance of oral communication in L2 learning (Levy & Razin, 2007; Wei & Xu, 2022). It has also been argued that learners having higher WTC are more likely to accomplish learning outcomes in the second language (Chu & Schallert, 2008). The contribution of WTC to L2 language learning may be attributed to the opportunities it provides for learners to entrench and strengthen the language. In line with this, Nation (2021) recommended utilizing meaning-focused output as a good means for vocabulary learning. WTC can foster and enhance the process of vocabulary learning as learners with higher WTC are more likely to engage themselves to meaning-focused output, which, in turn, helps them to know the words better.

Additionally, knowing a word has been found to involve both receptive/productive and size/depth facets (Masrai, 2021). It typically starts receptively mainly via reading and listening to increase the size of vocabulary knowledge; and then develops productively (Read, 2000) to enhance depth of word knowledge. Productive/depth mastery is, however, a slow and more challenging process than receptive/size mastery, in that learners should know detailed knowledge of words to be able to use them appropriately (Bloom, 2002; Hadley et al., 2018). To make this process occur faster and more effectively, learners need not only to start building up their depth vocabulary as early as possible but also to actively use words in their language production. They need to be willing to draw upon the words in their written or spoken communication. That said, WTC can be a great help to them in this regard, as it can function as a propeller that accelerates the process of using and knowing words faster and more profoundly.

Similarly, it has been recognized that to reach a deeper level of lexical knowledge, lexical items need to be used in different activities (Moir & Nation, 2002; Read & Dang, 2022). Nation and Ming-Tzu (1999), Nation (2022), and Waring and Takaki (2003) argue that vocabulary learning by reading-based approaches is not very effective as it would not cause learners to consciously try to internalize words. They suggest that an efficient approach for lexical learning should push learners to do conscious effort (that is, noticing) to learn the items meaningfully. It, in fact, implies that meaningful use of words (output hypothesis of Swain (1985) and their deep, conscious processing of words (in line with information processing hypothesis of Hulstijn’s (2001) would more help learners learn lexical words than merely committing a list of words to memory for which no or, at least, slight effective conscious effort is required. When learners use the items in various activities and contexts, they can notice subtler properties of lexical items, which, in turn, can foster their quality learning (Koizumi, 2005). One trait that can help learners to practice vocabulary is WTC, in that it augments the likelihood of frequent language use (Clément et al., 2007) and aids learners to learn lexical items efficiently and profoundly. Collectively, since increased involvement with a lexical item consolidates learners’ knowledge of that item, traits such as WTC substantially assist learners with deepening and strengthening their lexical command in their mental lexicon.

The findings of the current study align with those of Zhang and Lu (2015) reporting that strategies that deal with the associative meaning of words are better predictors of both breadth and depth vocabulary knowledge. Associative meaning refers to the interplay among vocabulary items in learners’ lexicon. Reaching a more complex interrelationship among the words in lexicon equates with reaching a deeper understanding of lexical knowledge. This complex interrelationship requires active use of words. Thus, WTC is also a critical factor here in that higher WTC leads to higher probability of practicing words.

Another point that can explain the contribution of WTC to depth of vocabulary knowledge is that high WTC learners are typically expected to be higher risk takers, compared with low WTC learners, who are willing to take part in an activity even if they may have no idea about (Heidari & Rashidi, 2019). In a similar vein, Norton and Toohey (2001) and Rubin and Thompson (1982) asserted that features such as being a risk taker, being motivated, and having positive attitudes may significantly result to successful learning outcomes. Thus, it stands to reason enough to ascribe these characteristics to high WTC learners in that they show more enthusiasm and energy to classroom activities. One important point to consider, however, is that WTC should not be taken a factor that directly impacts on deeper lexical learning of high WTC learners, as it has been proved to indirectly affect learning outcomes (Joe et al., 2017; Moir & Nation, 2002). Li et al. (2022) contended that WTC has not yet been reported to directly account for learning outcomes. It often triggers learners to become more engaged in classroom activities, which, in turn, increases the probability of learning.

## Conclusion

This study examined whether it is true to assert the higher the WTC, the higher depth of vocabulary knowledge for L2 learners. The obtained results of this study proved it to be true. This finding can be taken as a signpost for learners and teachers to achieve a deeper level of lexical knowledge. Being highly willing to communicate can be a great aid for learners to foster the quality of their lexical knowledge by strengthening their WTC and engaging themselves in varying activities both inside and outside classrooms. Teachers can also benefit from the results of this study, as their instruction can be more effective by seeking and utilizing methods and strategies that enhance their learners’ willingness to take part in classroom activities such that lexical items would be used more frequently and, consequently, are more efficiently and deeply entrenched in their learners’ mind. Teachers can, for instance, make use of group discussions or writing diaries activities in classes because these activities require high communication from learners and also push them to actively draw upon their word knowledge. Teachers can also motivate learners to form small groups of discussion out of classroom and try to utilize their learned words in their discussions.

This study was only a beginning step in response to the call for research on WTC in association with other language skills and domains, specifically vocabulary. To consolidate the present study findings, further replication studies are needed. Interested researchers are, then, recommended to carry out either conceptual or approximate replication studies to extend and confirm the present study findings. Additionally, carrying out studies to explore how WTC impacts on productive vocabulary knowledge is another potential avenue of research. Conducting studies in which other aspects, including but not limited to, multi-word expressions, proficiency differences, learning conditions, and cognitive styles are researched are also recommended. How differences in proficiency level, learning conditions (deliberate and incidental), and cognitive styles (field dependence/independence) may affect WTC and depth of vocabulary knowledge are also topics that should be researched to gain clearer understanding of WTC and depth of vocabulary knowledge.
